# P02.27. Martial arts exercise improves quality of life in overweight/obese premenopausal women

**DOI:** 10.1186/1472-6882-12-S1-P83

**Published:** 2012-06-12

**Authors:** Y Zhang, M Chyu, C Shen

**Affiliations:** 1 Texas Tech University Health Sciences Center, Lubbock, USA; 2 Graduate Healthcare Engineering Program, Texas Tech University, Lubbock, USA

## Purpose

Martial arts have been practiced as a physical activity for health and fitness and are recommended as such by the US Centers for Disease Control and Prevention. Previous studies showed that most forms of martial arts had positive effects on health. However, there is no data on the effects of martial arts exercise (MAE) on quality of life. This study evaluated the effects of 12 weeks of MAE intervention on quality of life among overweight/obese premenopausal women.

## Methods

Forty-seven premenopausal women were recruited and randomly assigned to either MAE or control group after matching the age and BMI. MAE curriculum (1 hr/session, 3 sessions/week for 12 weeks) features a non-competitive, non-contact, safe and fun personal/group exercise based on traditional martial arts training, covering a variety of techniques. Quality of life (QoL) was measured by the SF-36 form. Data were collected at baseline, 6, and 12 weeks. The analytic sample included 34 women who completed the study, 17 in each group. Data were analyzed using a repeated measures ANOVA model.

## Results

No statistically significant difference of QoL scores was found at baseline. After 12 weeks, the interaction of MAE and time significantly affected 6 of 8 QoL subscale scores including Role Physical, General Health, Vitality, Social Functioning, Role Emotional and Mental Health (p < 0.05) as well as the aggregated mental component (p< .01). MAE group participants reported significant improvements in those scales compared to those in the control group. MAE group tended to lose body weight (p=.09) along with decreased fat-free mass (p=.007) and muscle mass (p=.022).

## Conclusion

MAE may be a feasible, low-cost, and effective approach to improving QoL of overweight/obese premenopausal women. Our study underscores the need for further long-term studies using larger sample sizes to establish the benefits of MAE in this and other populations.

